# Temporal impact of hypothyroidism on porcine fetal growth and skeletal development

**DOI:** 10.14814/phy2.71080

**Published:** 2026-08-26

**Authors:** Kaylyn Rudy, Alyssa A. Smith, Brittany M. Wilson, Ryan Cabot, D. Rick Sumner, J. Scott Radcliffe, J. Alex Pasternak

**Affiliations:** ^1^ Department of Animal Sciences Purdue University West Lafayette Indiana USA; ^2^ Department of Animal and Food Sciences University of Kentucky Lexington Kentucky USA; ^3^ Department of Anatomy & Cell Biology Rush University Medical Center Chicago Illinois USA; ^4^ Department of Orthopedic Surgery Rush University Medical Center Chicago Illinois USA

**Keywords:** bone, hypothyroid, organ, phenotype, pig, thyroid hormone

## Abstract

In the fetal pig, circulating thyroid hormone and growth rate increase throughout gestation; however, the functional association between these coincident factors has not been established. We used a nonpathogenic model of induced fetal hypothyroidism, wherein pregnant gilts were treated with methimazole (MMI) for 21 days, starting on gestation days (GD) 34, 45, 55, or 65. The resulting fetal populations were deeply phenotyped, including body and organ weights, cranial and whole body morphometrics, radiography, and dual energy X‐ray absorptiometry (DXA) of the fore‐ and hindlimbs. MMI increased absolute fetal thyroid weight by 74%, 581%, 551%, and 315% on GD 55, 66, 76, and 86, respectively, indicating successful MMI‐induced fetal hypothyroidism at all time points. Crown rump length, girth, body weight, and the absolute weight of all fetal organs increased with GD, with no effects of hypothyroidism. However, MMI significantly reduced the length of the long bones in both fore‐ and hindlimbs by 11.1%–13.4% on GD 86, while bone area, as measured by DXA, decreased by 20%–21% for the same period. Collectively, these results indicate that overall fetal growth during mid to late gestation is not driven by thyroid hormones, but skeletal growth is negatively impacted by hypothyroidism in a temporally dependent manner.

## 1 INTRODUCTION

Thyroid hormones, including triiodothyronine (T3) and thyroxine (T4), are a critical part of the maternal‐fetal milieu, promoting fetal growth and the development of vital organ systems. A healthy human pregnancy is associated with a substantial upregulation in thyroid activity and a prerequisite uptick in the dietary requirement for iodine. Unfortunately, despite sustained global efforts to iodinate salt, a recent meta‐analysis indicates that just 47% of pregnant women receive sufficient iodine to meet this increased demand (Patriota et al., 2022). Even under conditions of iodine sufficiency, thyroid dysfunction is known to occur in cases of maternal/fetal thyroid pathology, abnormal nutritional plane, acute/chronic stress, and pathogenic infection. In developed countries such as the US, where the population is considered to be iodine sufficient, 0.3% of pregnant women are still found to be clinically hypothyroid, with an additional 2.45% classified as sub‐clinically hypothyroid (Klein et al., 1991). Such disruption is acutely associated with negative outcomes, increasing the likelihood of small for gestational age fetuses and low birth weight phenotypes (Derakhshan et al., 2020). The most severe cases of congenital hypothyroidism result in a cretin phenotype, characterized by short stature and neurological impairment (Bassett & Williams, 2016).

Much of our understanding with regards to the developmental consequences of hypothyroidism comes from rodent models of perinatal hypothyroidism, where maternal exposure to anti‐thyroid compounds such as methimazole (MMI) or propylthiouracil inhibit endogenous thyroid hormone activity in both the dam and fetus (Ghanbari & Ghasemi, 2017). In this model, maternal MMI treatment during gestation and lactation results in reduced offspring body weight, and an absolute and relative decrease in the weight of specific organs including the spleen and thymus (Nakamura et al., 2007). In addition, MMI‐induced gestational and lactational hypothyroidism altered craniofacial structure (Gecgelen Cesur et al., 2016), and reduced growth and ossification of the long bones (Ben Amara et al., 2012). The clinical relevance of this model is, however, limited by the altricial development observed in rats, and the prolonged pre‐ and postnatal suppression of thyroid hormone.

Swine are increasingly recognized as a high value biomedical model, owing to the high degree of anatomical and physiological similarities with humans (Lunney et al., 2021). In both humans and pigs, fetal thyroid hormone availability is primarily driven by activity in the fetal hypothalamic‐pituitary‐thyroid (HPT) axis, which becomes active in both species between 40% and 50% of the way through gestation (Brzezińska‐Slebodzińska & Slebodziński, 2004; Burrow et al., 1994). As in humans, thyroid independence in the fetal pig is maintained by a placental enzymatic barrier (Krysin et al., 1997), where maternal hormone is actively metabolized to liberate iodine for transport across the maternal fetal interface. Following activation, circulating T3 and T4 in the fetal pig progressively rise (Brzezińska‐Slebodzińska & Slebodziński, 2004) in conjunction with the exponential increase in fetal body and organ weights (McPherson et al., 2004). Undesirable disruption in this system is known to occur in the fetal pig in response to in utero infection (Pasternak et al., 2020), with modulation of the HPT axis and peripheral thyroid hormone metabolism consistent with type 1 allostasis (Ison et al., 2022; Smith et al., 2026). This effect is coincident with impaired skeletal ossification (Ko et al., 2022), and altered hyperplasia across multiple organ systems (Mulligan et al., 2022).

To test the hypothesis that developmental consequences of hypothyroidism on fetal growth and development are gestationally age dependent, the present study utilized a previously established model of MMI‐induced fetal hypothyroidism (Innis et al., 2026; Ison et al., 2023; Smith & Pasternak, 2025a). We used this model to produce fetal hypothyroidism in overlapping 3‐week periods starting prior to activation of the fetal HPT axis and spanning mid‐late gestation, and then evaluated the impact on fetal preservation, the accretion of fetal and organ specific mass, and skeletal development.

## 2 MATERIALS AND METHODS

### 2.1 Animal model

Phenotypic data and limb samples were derived from a previously published animal experiment (Smith & Pasternak, 2025a, 2025b), and the specific methods described in detail (Rudy, 2024). Briefly, a total of *n* = 24 commercial crossbred gilts were bred by artificial insemination using pooled Duroc semen, and pregnancy was confirmed via transcutaneous ultrasound around gestation day (GD) 30. Three to 5 days prior to the start of the experimental period, gilts were moved into individual gestation crates with fenceline contact and ad libitum access to water. From breeding through the end of this study, all gilts were maintained on a standard gestation diet that was formulated to meet or exceed dietary requirements as outlined by the National Research Council (2012), and was comprised primarily of corn, soybean, and dried distillers grains. Following an acclimation period, gilts were evenly split into two treatment groups, with *n* = 12 gilts receiving a daily oral dose of 5 mg/kg methimazole (MMI) (M8506, Sigma‐Aldrich, St. Louis, MO, USA) to induce fetal hypothyroidism, and *n* = 12 gilts receiving a daily sham control (CON). Within each of these groups, the *n* = 12 gilts were further split into four time points, with *n* = 3 gilts/group beginning treatment on GD 34, 45, 55, or 65 of the normal 114‐day gestation period, and each treatment period lasting for a total of 21 days. Throughout the treatment period, gilts were weighed twice weekly, and dosages adjusted accordingly. Additionally, maternal rectal temperatures were collected on this same schedule.

### 2.2 Fetal viability and organ weights

Following 21 days of treatment, all gilts were humanely euthanized to allow for fetal phenotyping and sampling on GD 55, 66, 76, and 86 for gilts beginning treatment on GD 34, 45, 55, or 65, respectively. Gilts were first stunned with a penetrating captive bolt and subsequently exsanguinated as per the AVMA guidelines (Leary, 2020), and the gravid uterus excised and linearized. Each uterine horn was opened along the anti‐mesometrial side and fetuses removed beginning at the uterotubal junction and working towards the cervix to preserve the relative position of each fetus and its corresponding placenta. Fetal sex was determined by examining the external genitalia, and fetal preservation determined as previously described (Jeon et al., 2026; Malgarin et al., 2021). In short, fetuses with an umbilical pulse were divided into the healthy viable or meconium stained classifications based on the presence or absence of yellowish‐brown material on the face or body. Fetuses without an observable umbilical pulse were subcategorized based on increasing levels of tissue degradation into either dead, autolyzed, or mummified categories. Maternal ovulation rate was determined by counting the number of corpora lutea present on both ovaries, and this count used in conjunction with both the total number of conceptuses and the number of viable conceptuses to determine embryonic and fetal survival, respectively. Following assessment of fetal preservation, each viable fetus was weighed and then dissected to collect the weights of individual organs, including the heart, liver, kidney, lung, spleen, brain, and thyroid. In addition to fetal assessment, the maternal thyroid was removed and weighed to assess the impact of MMI treatment on the dam. All animal procedures were carried out in compliance with Purdue University's animal care policies and approved by the Institutional Animal Care and Use Committee (IACUC Protocol #0123002344).

### 2.3 Fetal body and head morphometrics

Prior to dissection, all viable fetuses were imaged in left and right lateral recumbency, along with reference size markers. A selection of morphometric parameters was then assessed using a semi‐automated analysis pipeline in ImageJ (Schneider et al., 2012), as previously described (Jeon et al., 2025). Briefly, crown rump length was measured as the distance from the base of the tail, moving along the back, and ending at the inflection point between the snout and forehead. Girth was measured across the chest immediately caudal to the front limb and perpendicular to the central axis of the body. Finally, the shape of the skull was assessed through measurement of the skull height, snout height, skull linear length, and skull curved length. Skull linear length and curved length were assessed as the linear and curved distance, respectively, from the tip of the snout to the external occipital protuberance. Snout height was measured from the crown to the base of the jaw going along the crease of the snout. Skull height was measured by drawing a line from in front of the jowl to the top of the skull such that the line touched the caudal edge of the eye opening. For each parameter, measurements from the left and right side of each fetus were considered technical replicates and used to calculate Pearson's correlation coefficients (*r*) and coefficients of variance (CV) before averaging values for each fetus for later statistical assessment.

### 2.4 Fetal skeletal radiographic analysis

Within each litter, left and right fetal fore‐ and hindlimbs were isolated from the centermost viable male and female fetuses from each uterine horn and frozen in plastic bags at −20°C for later assessment (*n* = 96 fetuses), with this methodology previously described (Ison et al., 2023). Briefly, radiographs were taken of the frozen limbs, and the resultant images were analyzed using a custom semiautomated image analysis pipeline in ImageJ (Schneider et al., 2012), with assessed parameters including the lengths of the tibia, fibula, radius, ulna, femur, humerus, and scapula, along with the width of the widest portion of the scapula. To evaluate the accuracy and repeatability of each measurement, the coefficient of determination (*R* ^2^) and coefficient of variance (CV) for each parameter were calculated using measurements from the left and right leg before results were averaged within fetus for later statistical assessment.

### 2.5 Fetal dual‐energy X‐ray absorptiometry (DXA)

Fore‐ and hindlimbs were thawed and scanned by DXA (Kubtec Parameter 3D Cabinet X‐ray System with Digimus® with bone mineral density analysis software) for whole limb bone area, bone mineral content, and bone mineral density. Scans were acquired at energies of 25 kV and 40 kV. Skin was removed but all muscles were left intact. Forelimbs included scapulae and all bones distal, while hindlimbs included the femurs and all bones distal.

### 2.6 Statistical analyses

Maternal level effects including body weight, rectal temperature, and thyroid weight were evaluated using a linear model with treatment and time as fixed effects. Fetal body and organ weights, morphometrics, and radiology measurements were all initially evaluated using a linear mixed effects model using the package nlme (Pinheiro et al., 1999), including treatment, time, sex, and the interaction as fixed effects and gilt as a random effect. Sex and its interactions were nonsignificant for any parameter measured and were thus removed from subsequent analyses. Model residuals for all morphometric and the majority of radiology measurements were found to be normally distributed as assessed using qqplots. In contrast, absolute and relative organ weights required log normalization to achieve normally distributed residuals, while bone mineral content in the forelimb required reciprocal transformation as identified by Box‐Cox (Box & Cox, 1964). Estimated marginal means (aka least square means) were generated using the emmeans package (Lenth & Piaskowski, 2017) and pairwise comparisons made using the Dunn–Šidák correction for multiple testing. Relationships between morphological parameters were evaluated using Pearson's correlation. Outcomes are plotted using the ggplot2 package (Wickham, 2016) in the form of estimated marginal means with 95% confidence intervals. The threshold for statistical significance was *p* < 0.05.

## 3 RESULTS

### 3.1 Maternal and litter level effects

MMI had no significant effect on maternal weight gain (*p* = 0.262) or rectal temperature (*p* = 0.54) over the course of the 21‐day treatment periods. Additionally, maternal thyroid weights at necropsy did not differ (*p* = 0.160) between treatment groups (data not shown). Total ovulation rate, based on the observed number of corpus luteum, ranged from 12 to 23 (Table 1) and was not altered by treatment. The overall average litter size at necropsy was 14.16 and was not significantly affected by treatment (*p* = 0.12), resulting in a total fetal population of 340. The overwhelming majority of fetuses (335) were deemed viable at necropsy (Table 1), with the remaining five nonviable fetuses found to be mummified. Of the mummified fetuses, three came from CON litters with one each on GD 66, 76, and 86, and the remaining two found in 1 L from an MMI treated gilt on GD 86. As such, there was no discernable effect of treatment on embryonic (*p* = 0.139) or fetal loss (*p* = 0.126). As the stage of gestation at which each mummified fetus was lost could not be determined, and the degree of decay compromised the accuracy of in‐depth phenotyping, they were subsequently removed from all remaining analyses.

**TABLE 1:** Total fetal count and preservation status on gestation days (GD) 55, 66, 76, and 86 following 21 days of maternal treatment with 5 mg/kg/day of methimazole or an equivalent sham control.

GD	Group	Fetal preservation status					Total fetuses	Corpus luteum (#)	Embryonic survival (%)	Fetal survival (%)
		Viable	Meconium stained	Dead	Autolyzed	Mummified				
55	CON	50	0	0	0	0	50	19.33 (17–21)	86.32	86.32
	MMI	47	0	0	0	0	47	19 (17–21)	82.34	82.34
66	CON	32	0	0	0	1	33	19 (17–22)	58.39	56.87
	MMI	41	0	0	0	0	41	16.67 (15–18)	82.64	82.64
76	CON	46	0	0	0	1	47	19.33 (15–22)	81.63	80.04
	MMI	44	0	0	0	0	44	15.33 (14–17)	96.08	96.08
86	CON	35	0	0	0	1	36	15 (12–19)	77.44	75.69
	MMI	40	0	0	0	2	42	18.67 (16–23)	74.86	71.96
Total		335	0	0	0	5	340	17.79 (12–23)	79.96	78.99

### 3.2 Fetal body and organ growth

**FIGURE 1:**
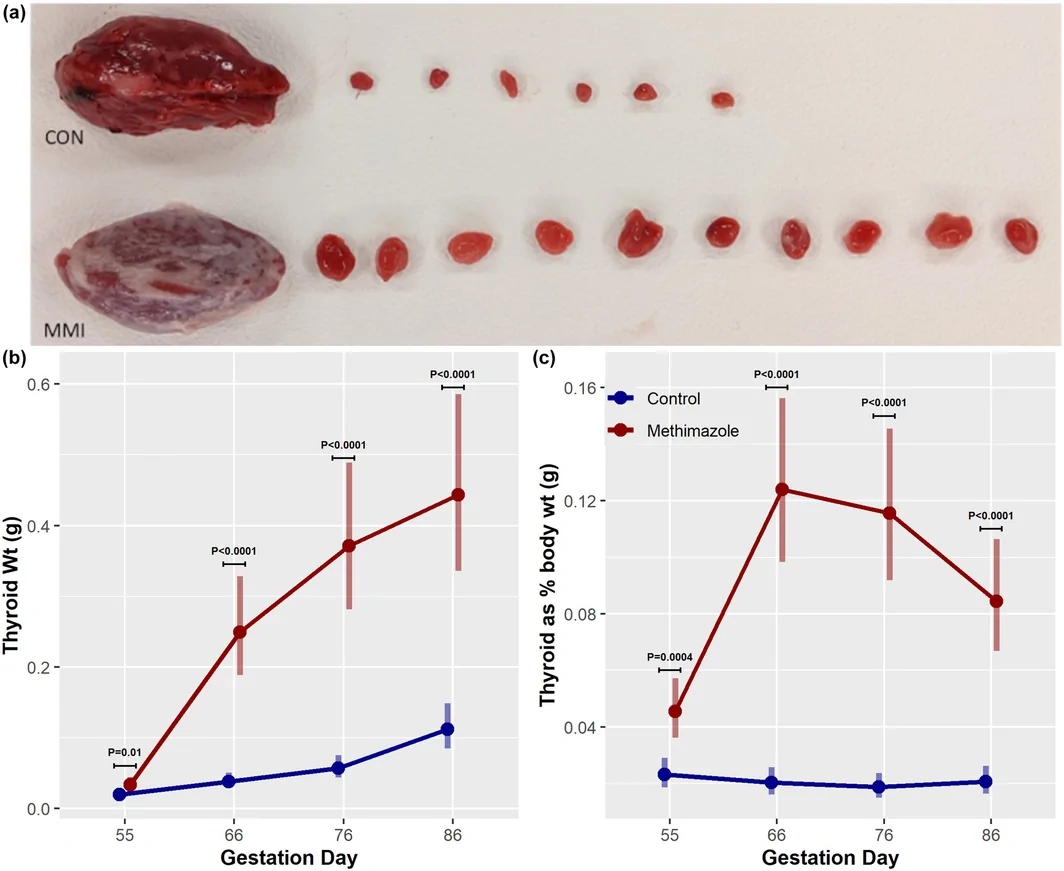
Representative image (a) of maternal and fetal thyroids collected from control and 5 mg/kg/day methimazole litters at gestation day 66. Estimated marginal means and 95% confidence intervals for the absolute (b) and relative weight (c) of fetal thyroids collected on gestation days 55, 66, 76, and 86 following 21 days of maternal treatment with methimazole or an equivalent sham control. Statistically significant differences between treatment groups within each time point were derived from a linear mixed effects model including treatment by time as fixed effects and dam as a random effect.

The absolute and relative weights of the thyroid increased at all four time points, indicating a successful induction of hypothyroidism in the fetuses from MMI treated gilts (Figure 1). Thyroid parameters showed a significant treatment by time interaction, with an increase in absolute weight of 0.014, 0.211, 0.314, and 0.332 g on GD 55, 66, 76, and 86, respectively. Unsurprisingly, fetal body weight significantly increased with each 10‐day increment in gestational age (Table 2). Similarly, the absolute weight of all organs, including heart, liver, lung, kidney, spleen, and brain, increased with gestational age. The absolute weight of these organs was not significantly altered by thyroid status at any time point, though the absolute weight of the liver approached statistical significance (*p* = 0.087) (Table 2). Relative weights of the liver and kidney significantly decreased in a stepwise fashion with gestational age, with MMI significantly increasing relative liver weight on GD 77 (*p* = 0.014). A similar numerical increase occurred at GD 66, but did not reach statistical significance (*p* = 0.061). The relative weight of the lung numerically decreased from GD 55 onward, with the difference approaching significance on GD 76 (*p* = 0.075) and reaching significance on GD 86 (*p* = 0.007). The relative weight of the heart was also significantly reduced between GD 55 and 66 but remained stable thereafter and was not affected by treatment. The relative weight of the spleen significantly increased in a stepwise fashion from GD 55 onward, with the effect of MMI approaching significance (*p* = 0.054). Finally, the relative weight of the brain remained largely stable over the time course evaluated, with significant differences observed between GD 66 and 86 only. Consequently, the brain to liver weight ratio increased over time, with the effect of MMI approaching significance on GD 66 (*p* = 0.079) and 76 (*p* = 0.067).

**TABLE 2:** Estimated marginal means and 95% confidence intervals for fetal body weight and the absolute and relative weight of key fetal organs collected on gestation days 55, 66, 76, and 86 following 21 days of maternal treatment with methimazole or an equivalent sham control.

	Organ	55		66		76		86		*p*		
		Control	Methimazole	Control	Methimazole	Control	Methimazole	Control	Methimazole	Treatment	Time	Interaction
Absolute Weight	Body	84.325 (74.767–95.105)^a^	73.35 (64.788–83.044)^a^	187.224 (164.353–213.278)^b^	200.865 (177.101–227.817)^b^	302.811 (267.384–342.933)^c^	320.948 (283.263–363.646)^c^	536.049 (470.481–610.755)^d^	525.731 (463.166–596.748)^d^	0.921	*p* < 0.001	0.296
	Heart	0.67 (0.597–0.753)^a^	0.619 (0.549–0.699)^a^	1.347 (1.187–1.529)^b^	1.456 (1.289–1.646)^b^	2.058 (1.824–2.322)^c^	2.343 (2.076–2.645)^c^	3.939 (3.469–4.472)^d^	3.66 (3.235–4.14)^d^	0.536	*p* < 0.001	0.206
	Liver	5.099 (4.351–5.974)^a^	4.703 (3.995–5.536)^a^	6.965 (5.887–8.24)^b^	8.394 (7.121–9.896)^b^	8.818 (7.489–10.383)^c^	10.905 (9.258–12.845)^c^	14.12 (11.929–16.713)^d^	15.113 (12.808–17.832)^d^	0.087	*p* < 0.001	0.251
	Lung	2.894 (2.597–3.225)^a^	2.54 (2.271–2.841)^a^	5.931 (5.261–6.686)^b^	6.384 (5.695–7.157)^b^	9.528 (8.515–10.661)^c^	9.344 (8.345–10.461)^c^	15.848 (14.06–17.862)^d^	14.366 (12.797–16.128)^d^	0.429	*p* < 0.001	0.279
	Kidney	1.395 (1.168–1.667)^a^	1.215 (1.013–1.459)^a^	2.433 (2.016–2.937)^b^	2.629 (2.186–3.161)^b^	3.267 (2.721–3.923)^c^	3.74 (3.113–4.492)^c^	5.504 (4.555–6.651)^d^	5.963 (4.952–7.181)^d^	0.486	*p* < 0.001	0.427
	Spleen	0.03 (0.024–0.038)^a^	0.022 (0.018–0.028)^a^	0.111 (0.087–0.142)^b^	0.086 (0.068–0.109)^b^	0.304 (0.241–0.384)^c^	0.256 (0.202–0.324)^c^	0.656 (0.512–0.84)^d^	0.704 (0.554–0.894)^d^	0.077	*p* < 0.001	0.381
	Brain	2.272 (2.104–2.454)^a^	2.141 (1.978–2.317)^a^	4.873 (4.495–5.284)^b^	4.889 (4.515–5.294)^b^	8.852 (8.178–9.581)^c^	9.065 (8.374–9.813)^c^	16.108 (14.855–17.468)^d^	15.243 (14.072–16.511)^d^	0.551	*p* < 0.001	0.611
Relative Weight	Heart	0.795 (0.734–0.862)^a^	0.844 (0.777–0.918)^a^	0.719 (0.659–0.786)^b^	0.725 (0.666–0.789)^b^	0.68 (0.625–0.739)^b^	0.73 (0.671–0.794)^b^	0.735 (0.673–0.803)^b^	0.698 (0.641–0.76)^b^	0.444	0.005	0.421
	Liver	6.046 (5.576–6.555)^a^	6.409 (5.897–6.966)^a^	3.725 (3.415–4.063)^b^	4.179 (3.841–4.547)^b^	2.912 (2.679–3.166)	3.397 (3.125–3.694)^c^*	2.617 (2.399–2.855)^d^	2.877 (2.642–3.132)^d^	0.003	*p* < 0.001	0.677
	Lung	3.432 (3.1–3.799)^a^	3.467 (3.123–3.85)^a^	3.164 (2.841–3.522)^ab^	3.179 (2.861–3.532)^ab^	3.15 (2.837–3.498)^ab^	2.909 (2.619–3.231)^ab^	2.97 (2.666–3.307)^b^	2.738 (2.463–3.044)^b^	0.31	0.01	0.674
	Kidney	1.655 (1.512–1.811)^a^	1.656 (1.509–1.818)^a^	1.302 (1.181–1.435)^b^	1.309 (1.191–1.438)^b^	1.079 (0.983–1.184)^c^	1.165 (1.061–1.279)^c^	0.998 (0.904–1.101)^c^	1.12 (1.018–1.232)^c^	0.164	*p* < 0.001	0.525
	Spleen	0.036 (0.029–0.044)^a^	0.03 (0.024–0.038)^a^	0.059 (0.047–0.074)^b^	0.043 (0.034–0.053)^b^	0.1 (0.081–0.125)^c^	0.08 (0.064–0.099)^c^	0.123 (0.097–0.154)^d^	0.134 (0.107–0.168)^d^	0.054	*p* < 0.001	0.283
	Brain	2.694 (2.439–2.975)^ab^	2.918 (2.634–3.234)^ab^	2.607 (2.339–2.906)^a^	2.434 (2.193–2.702)^a^	2.926 (2.639–3.243)^ab^	2.824 (2.546–3.132)^ab^	3.012 (2.702–3.358)^b^	2.902 (2.611–3.224)^b^	0.678	0.028	0.464

*Note*: Unique letter superscripts denote statistically significant (*p* < 0.05) changes over time and * indicates statistically significant differences between treatment groups within each time point, as identified by a linear mixed effects model including treatment by time as fixed effects and dam as a random effect.

### 3.3 Fetal morphometrics

**FIGURE 2:**
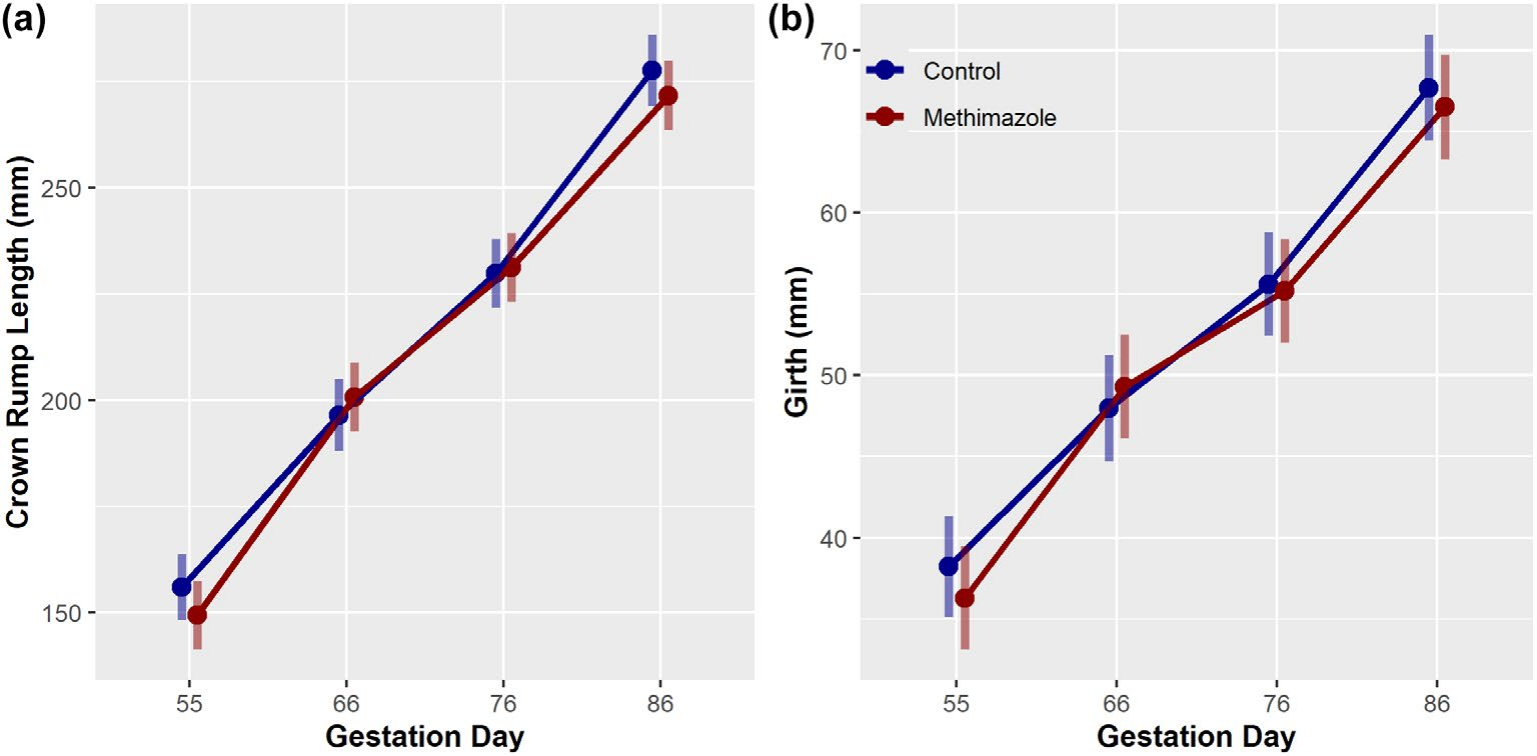
Estimated marginal means and 95% confidence intervals for fetal crown rump length (a) and girth (b) on gestation days 55, 66, 76, and 86 following 21 days of maternal treatment with methimazole or an equivalent sham control.

**FIGURE 3:**
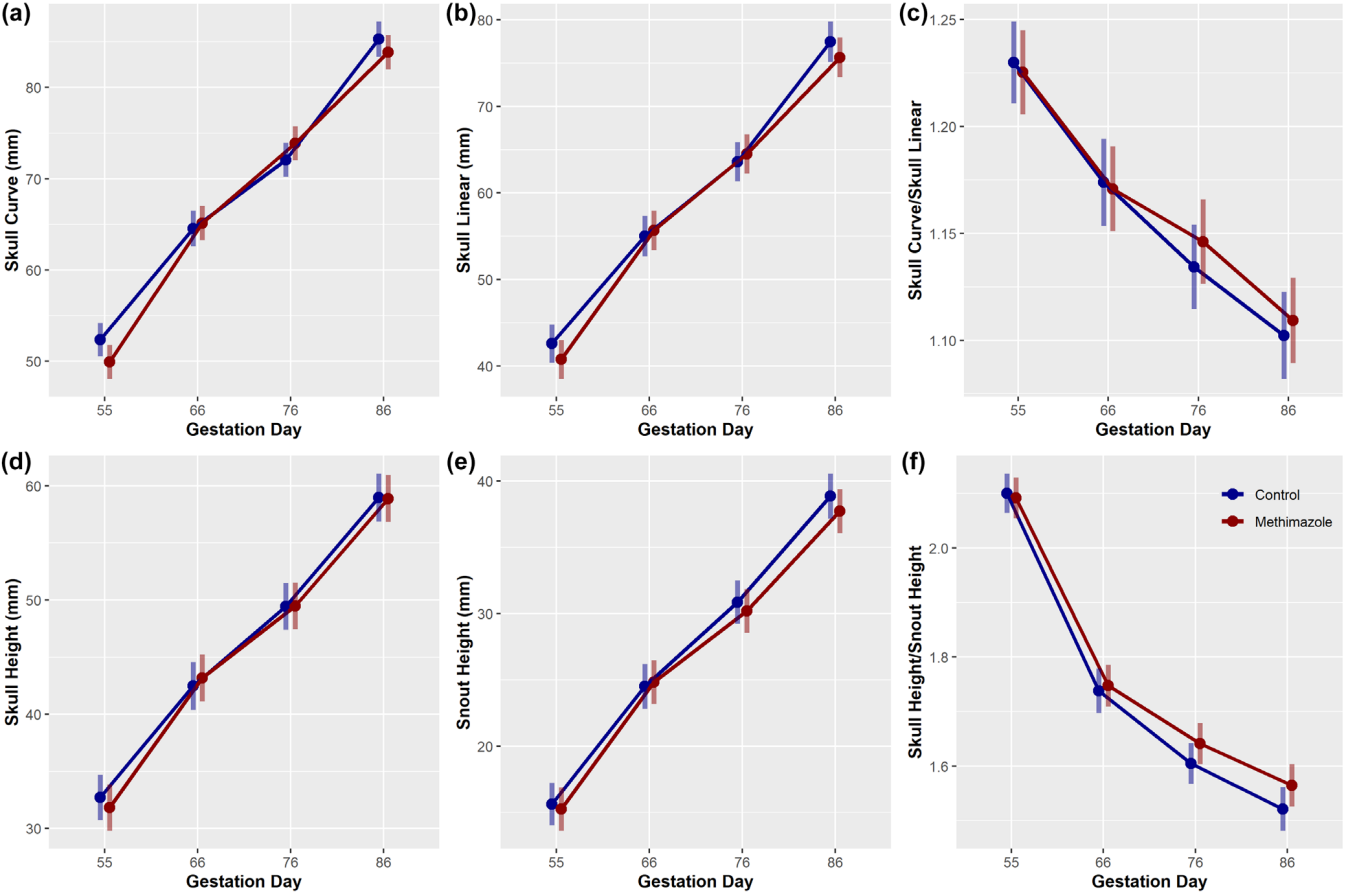
Estimated marginal means and 95% confidence intervals for measurements of length of the skull curvature (a), linear length of the skull (b), the ratio of skull lengths (c), skull height (d), snout height (e), and the ratio of skull to snout height (f) as measured on fetuses in lateral recumbency on gestation days 55, 66, 76, and 86 following 21 days of maternal treatment with methimazole or an equivalent sham control. No significant impacts of treatment were observed on any of the measured parameters.

To establish the impact of in utero hypothyroidism on fetal morphology, we first assessed standard metrics including crown rump length and girth using images of each fetus in left and right lateral recumbency. The CV between left and right measures of each fetus was 2.05% and 1.47% for crown rump length and girth, respectively, with regression coefficients (*R* ^2^) over time exceeding 0.97. Both measures significantly increased with gestational age, but were not significantly different between treatment groups (Figure 2). We further evaluated the impact of MMI treatment on cranial morphometrics (Figure 3). Both skull curvature and the linear equivalent increased with gestational age, while the ratio of the two significantly decreased. Similarly, skull and snout height increased with gestational age, while the ratio of skull over snout height decreased. None of the cranial morphometrics were significantly impacted by fetal thyroid status. Interestingly, there was a significant correlation between brain to liver weight ratio and both the ratios of skull curvature to linear length (*ρ* = −0.54, *p* < 0.001) and skull height to snout height (*ρ* = −0.71, *p* < 0.001).

### 3.4 Fetal bone growth

**FIGURE 4:**
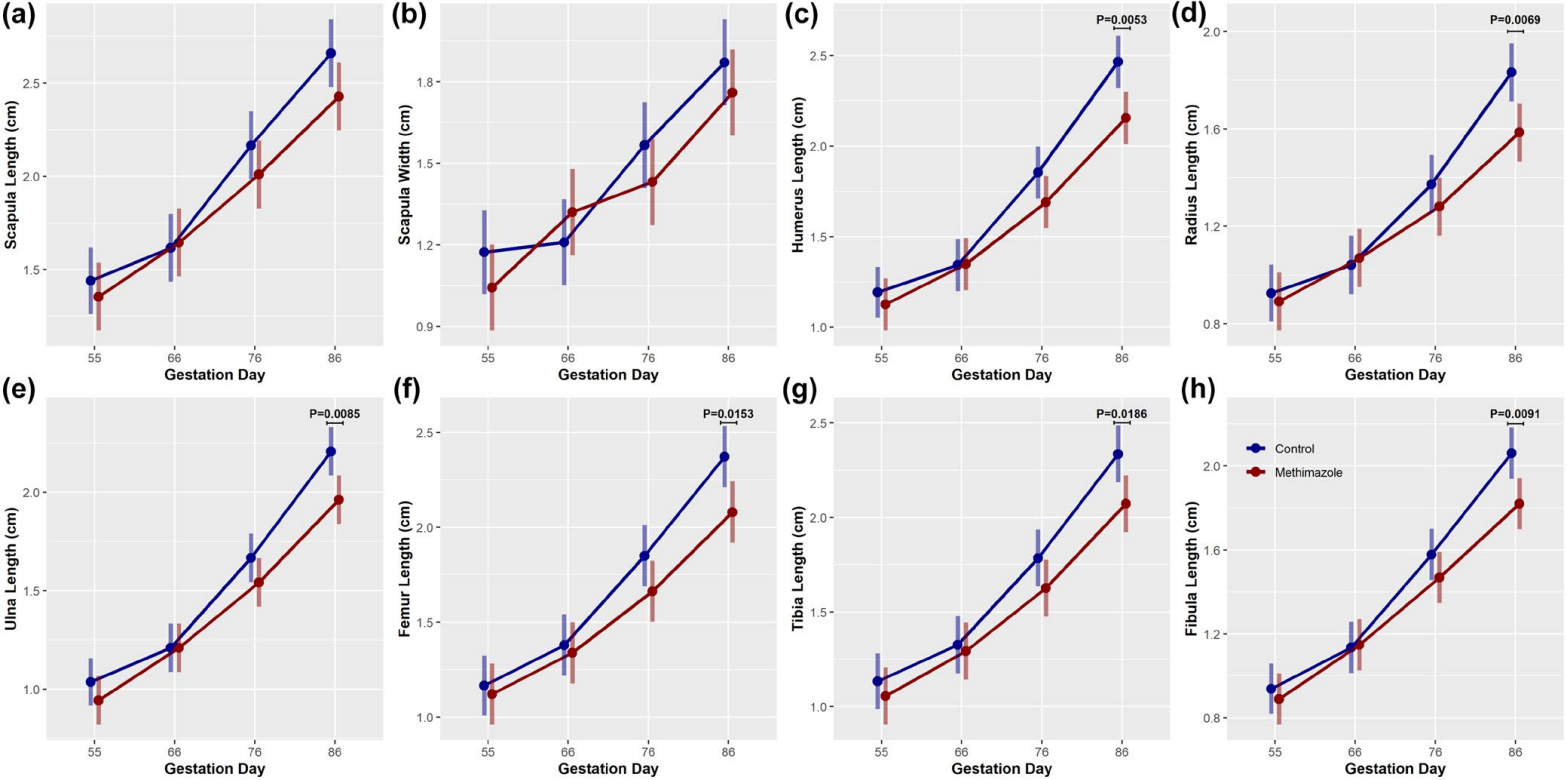
Estimated marginal means and 95% confidence intervals for the width and length of bones measured from radiographs of the fetal fore‐ and hindlimbs collected on gestation days 55, 66, 76, and 86 following 21 days of maternal treatment with methimazole or an equivalent sham control.

**FIGURE 5:**
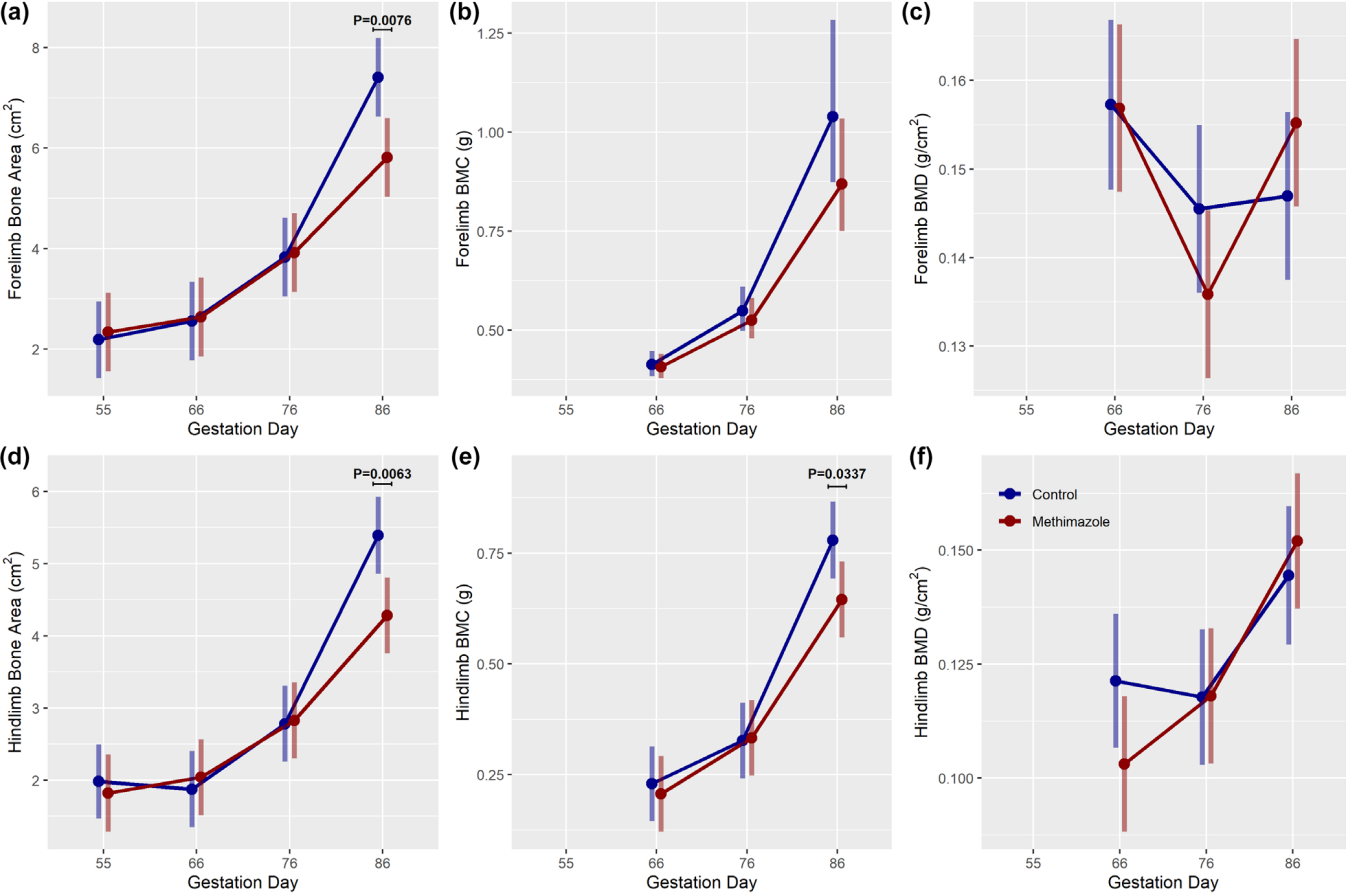
Estimated marginal means and 95% confidence intervals for bone area, mineral content, and density as determined by Dual‐energy X‐ray Absorptiometry (DXA) in fetal fore‐ (a–c) and hindlimbs (d–f) collected on gestation days 55, 66, 76, and 86 following 21 days of maternal treatment with methimazole or an equivalent sham control.

The impact of fetal hypothyroidism on bone development was assessed in the left and right fore‐ and hindlimbs from a subset of 96 fetuses split evenly across gestational age and treatment group (Figure 4). The CV for all measurements from the left and right side averaged 2.78%, with the largest variation observed for scapula width (CV = 3.251%) and fibula length (CV = 3.633%). The regression coefficient (*R* ^2^) for left and right bone measures over time exceeded 0.91 for all measurements. The length and width of the scapula and the length of the humerus, radius, and ulna all increased significantly (*p* < 0.001) GD day 55 to GD 86 of gestation (Figure 4). There was no significant effect of hypothyroidism on scapula width (*p* = 0.225), while the difference in scapula length approached significance (*p* = 0.086). In contrast, 21 days of fetal hypothyroidism resulted in a significant reduction in the length of the humerus (*p* = 0.005), radius (*p* = 0.007), and ulna (*p* = 0.009) in limbs collected on GD 86. The length of each long bone measured in the hind limb, including femur, tibia, and fibula, significantly increased from GD 55 to GD 86 (Figure 4). The length of the femur was significantly reduced by hypothyroidism in samples collected on GD 86 (*p* = 0.015), while the numerical reduction observed at GD 76 did not reach statistical significance (*p* = 0.100). The length of the fibula (*p* = 0.009) and tibia (*p* = 0.019) were also significantly reduced on GD 86. Bone mineral content and by extension mineral density were below the threshold of detection (0.2 g) in samples collected on GD 55, and this timepoint was removed from subsequent statistical analyses. Bone area and bone mineral content and bone mineral density increased with gestational age (*p* < 0.001). MMI treatment had no significant impact on any DXA parameter in samples from GD 55–76 (Figure 5). However, MMI treatment significantly reduced bone area in both fore (*p* = 0.008) and hind limb (*p* = 0.007) in fetuses evaluated on GD 86 (Figure 5). Bone mineral content on GD 86 was also significantly reduced by MMI treatment in the hind (*p* = 0.034) but not forelimb (*p* = 0.139).

## 4 DISCUSSION

In the fetal pig, the hypothalamic–pituitary‐thyroid (HPT) axis is initially activated around GD 45 (Brzezińska‐Slebodzińska & Slebodziński, 2004), with this early activation thought to be critical in swine due to the existence of a placental enzymatic barrier that metabolizes maternal thyroid hormones to biologically inactive forms as they move across the maternal‐fetal interface (Krysin et al., 1997). Following activation of this system, activity within the fetal HPT axis is continually upregulated, resulting in an exponential increase in circulating thyroid hormone levels as gestation progresses (Brzezińska‐Slebodzińska & Slebodziński, 2004). Based on studies in other species, this increase in circulating thyroid hormone levels is thought to play a number of critical roles in fetal development, including contributing to the accretion of fetal mass and the maturation of vital organs (Forhead & Fowden, 2014). To better understand the role of thyroid hormones in the development and maturation of the fetal pig throughout gestation, we utilized a temporal model of fetal hypothyroidism achieved via maternal administration of MMI. In addition to the present increases in fetal thyroid weights indicating the establishment of goiter in MMI fetuses, the successful induction of hypothyroidism in these fetuses was previously reported (Smith & Pasternak, 2025a). In this prior report, we showed that MMI caused goitrous thyroid histology and significantly reduced circulating T4 in these fetuses at all four time points, while T3 was significantly reduced on GD 76 and 86 (Smith & Pasternak, 2025a). Collectively, these results demonstrate that MMI fetuses experience severe systemic fetal hypothyroidism and goiter.

As with our prior study involving the treatment of pregnant pigs with MMI during late gestation (Ison et al., 2023), we observed no apparent increase in the weight of the dam's thyroid gland, indicating that a daily dose of 5 mg/kg of MMI, over a 21 day period, is not sufficient to induce maternal hypothyroidism. Recent studies involving the use of MMI in postnatal pigs have demonstrated that while a dose of 10 mg/kg/day is sufficient to induce a significant increase in thyroid weight and severe goitrous histology, it results in only mild systemic hypothyroidism (Fazioli et al., 2024). This differs from historical studies which found that a dose of 2 mg/kg/day of MMI was sufficient to induce severe suppression of the HPT axis in postnatal pigs (Ingram & Dauncey, 1986), a difference which led Fazioli et al. to suggest that intensive selection over the intervening 38 years has created a robust central resistance to hypothyroidism in contemporary pigs. Interpreted in conjunction with recent reports, the lack of effect on maternal thyroid weight observed in the present study may suggest that even a partial reduction in the daily dosage of MMI renders the treatment ineffective. Alternatively, the Type II allostasis within the HPT axis typically associated with pregnancy (Chatzitomaris et al., 2017) may further augment the dam's resistance to pharmacological induction of hypothyroidism.

Interestingly, the exponential increases in circulating fetal thyroid hormone levels previously described in the fetal pig (Brzezińska‐Slebodzińska & Slebodziński, 2004) mirror the exponential growth observed during the second half of gestation (McPherson et al., 2004). Based primarily on ovine models involving fetal thyroidectomy, it has been proposed that thyroid hormones are critical for the accretion of fetal mass (Forhead & Fowden, 2014), however, this cause and effect relationship remains understudied in swine. In an attempt to better understand the apparent relationship between fetal growth and thyroid hormone abundance in the pig, we evaluated the effects of 21‐day periods of fetal hypothyroidism induced at 10‐day intervals between mid to late gestation. In contrast to previous hypotheses, the results of the present study indicate that fetal hypothyroidism has minimal effects on the accretion of fetal or organ mass in the pig, except for the appendicular skeleton.

This lack of change in fetal weight observed in the present study is similar to results seen in rats with MMI‐induced hypothyroidism (Mallela et al., 2014). It is further consistent with early descriptions of the “hairless pig malady”, where maternal dietary iodine deficiency negatively impacted fetal development and postnatal survival but not body weight (Hart & Steenbock, 1918). However, more recent studies investigating the effects of viral infection‐induced maternal and fetal hypothyroidism during late gestation observed a significant downregulation of cell division across seven fetal organs (Mulligan et al., 2022). While the effects on cell cycle regulation in infected, hypothyroid pigs could be the result of viral infection rather than a direct effect of endocrine disruption, subsequent studies using a non‐pathogenic model of hypothyroidism also show some degree of cell cycle dysregulation in the hypothyroid fetal pig (Smith et al., 2024). The incongruity between this previously described hypothyroidism‐induced decrease in cellular hyperplasia and the absence of an effect on fetal or organ mass may suggest a compensatory response in the form of hypertrophy. Alternatively, the 21 days of fetal hypothyroidism may not be sufficient to observe a meaningful gross impact. Thus, future studies should investigate the impact of extended periods of fetal hypothyroidism and the relative role of hyperplasia and hypertrophy in the thyroid hormone regulated accretion of fetal mass.

The MMI fetuses in the present study also showed no significant variation in crown rump length, girth, or skull measurements when compared to age‐matched CON fetuses. While the present lack of an effect on crown rump length is consistent with our prior work, when fetal hypothyroidism was produced between GD 85 and 106, it resulted in a significant increase in fetal girth (Ison et al., 2023). The incongruity in these morphometric results observed during late gestation led the previous authors to hypothesize that the increase in girth was the result of increased subcutaneous fat deposition (Ison et al., 2023). Although not directly measured in the present study, the absence of non‐allometric growth may suggest that fat deposition is not affected during earlier stages of gestation.

In the human, fetal hypothyroidism has long been associated with detrimental developmental effects such as intrauterine growth restriction and cretinism. Cretinism is used to describe a unique human birth phenotype associated with congenital hypothyroidism, which includes severe neurological damage and growth retardation in humans (Lauffer et al., 2021; Salisbury, 2003). The present study found no significant impact of hypothyroidism on the absolute or relative weight of the fetal brain; however, this does not preclude a developmental impact that could negatively impact postnatal cognitive function. Thus, future studies should evaluate behavioral outcomes in piglets following in utero MMI exposure to determine if the effects of fetal hypothyroidism on human neurological development can be replicated in swine.

A second defining characteristic of cretinism is a decrease in stature resulting from reduced in utero bone development. Thyroid hormone is essential for fetal bone growth throughout gestation, with previous studies in thyroidectomized fetal sheep reporting an impact of hypothyroidism on bone structure (Lanham et al., 2011). In the present study, MMI treatment between GD 65 and 86 of gestation resulted in significantly shorter lengths of the humerus, radius, ulna, femur, tibia, and fibula, while a similar numerical effect in the treatment period terminating 10 days earlier did not reach statistical significance. The impact of hypothyroidism on bone growth during this specific window is further supported by DXA, which indicated a significant reduction in bone area and bone mineral content at GD 86. However, when the bone mineral content was normalized to skeletal size (i.e., the bone mineral density), the negative effect of MMI treatment was no longer apparent. Interestingly, negative effects of MMI on bone size and bone mineral content were not observed at any of the earlier study time periods. Bone length was not found to be reduced in a previous study conducted in late gestation (GD 85–106) swine (Ison et al., 2023). Collectively, these results indicate that the effect of thyroid hormones on bone growth may be restricted to specific developmental periods.

## 5 CONCLUSIONS

Overall, our results show that MMI‐induced hypothyroidism has no apparent impact on fetal survival or body weight during mid to late gestation, with only a minimal impact observed on the growth of key fetal organs. This indicates that short duration fetal hypothyroidism does not have a significant impact on the accretion of fetal body or organ mass, and thyroid hormones may not be a major factor regulating fetal growth during this time period in the pig. In contrast to body and organ growth, hypothyroidism induced between GD 65–86 caused a significant reduction in the length, area, and bone mineral content of long bones, indicating a temporal influence of thyroid hormones in regulating skeletal development.

## AUTHOR CONTRIBUTIONS

**Kaylyn Rudy:** Investigation. **Alyssa A. Smith:** Formal analysis; investigation; methodology. **Brittany M. Wilson:** Investigation; methodology. **Ryan Cabot:** Conceptualization; funding acquisition; supervision. **D. Rick Sumner:** Funding acquisition; methodology. **J. Scott Radcliffe:** Conceptualization; funding acquisition; methodology; project administration. **J. Alex Pasternak:** Conceptualization; formal analysis; funding acquisition; investigation; methodology; project administration; supervision; visualization.

## FUNDING INFORMATION

This work is supported by the Foundational and Applied Science Program, project award no. 2023‐67015‐45866, from the U.S. Department of Agriculture's National Institute of Food and Agriculture and Grant Number R21HD102026 from the National Institutes of Health.

## CONFLICT OF INTEREST STATEMENT

The authors declare no conflict of interest.

## Data Availability

The data that support the findings of this study are available from the corresponding author upon reasonable request.

## References

[phy271080-bib-0001] Bassett, J. H. D. , & Williams, G. R. (2016). Role of thyroid hormones in skeletal development and bone maintenance. Endocrine Reviews, 37, 135–187. 10.1210/er.2015-1106 26862888 PMC4823381

[phy271080-bib-0002] Ben Amara, I. , Troudi, A. , Soudani, N. , Guermazi, F. , & Zeghal, N. (2012). Toxicity of methimazole on femoral bone in suckling rats: Alleviation by selenium. Experimental and Toxicologic Pathology, 64, 187–195. 10.1016/j.etp.2010.08.005 20833006

[phy271080-bib-0003] Box, G. E. P. , & Cox, D. R. (1964). An analysis of transformations. Journal of the Royal Statistical Society, Series B: Statistical Methodology, 26, 211–243. 10.1111/j.2517-6161.1964.tb00553.x

[phy271080-bib-0004] Brzezińska‐Slebodzińska, E. , & Slebodziński, A. B. (2004). Changes in thyroxine, 3,3′,5‐triiodothyronine and 3,3′5’‐triiodothyronine content in the thyroid gland and in serum to thyroid tissue iodothyronine ratios during ontogenesis in the fetal pig. Acta Veterinaria Hungarica, 52, 379–387. 10.1556/AVet.52.2004.4.1 15595272

[phy271080-bib-0005] Burrow, G. N. , Fisher, D. A. , & Larsen, P. R. (1994). Maternal and fetal thyroid function. New England Journal of Medicine, 331, 1072–1078. 10.1056/NEJM199410203311608 8090169

[phy271080-bib-0006] Chatzitomaris, A. , Hoermann, R. , Midgley, J. E. , Hering, S. , Urban, A. , Dietrich, B. , Abood, A. , Klein, H. H. , & Dietrich, J. W. (2017). Thyroid allostasis‐adaptive responses of thyrotropic feedback control to conditions of strain, stress, and developmental programming. Frontiers in Endocrinology, 8, 163. 10.3389/fendo.2017.00163 28775711 PMC5517413

[phy271080-bib-0007] Derakhshan, A. , Peeters, R. P. , Taylor, P. N. , Bliddal, S. , Carty, D. M. , Meems, M. , Vaidya, B. , Chen, L. , Knight, B. A. , Ghafoor, F. , Popova, P. V. , Mosso, L. , Oken, E. , Suvanto, E. , Hisada, A. , Yoshinaga, J. , Brown, S. J. , Bassols, J. , Auvinen, J. , … Korevaar, T. I. M. (2020). Association of maternal thyroid function with birthweight: A systematic review and individual‐participant data meta‐analysis. The Lancet Diabetes and Endocrinology, 8, 501–510. 10.1016/S2213-8587(20)30061-9 32445737 PMC8168324

[phy271080-bib-0008] Fazioli, J. C. , Mulligan, M. K. , Ison, E. K. , & Pasternak, J. A. (2024). Impact of methimazole‐induced hypothyroidism on postnatal swine. Physiological Reports, 12, e16007. 10.14814/phy2.16007 38658325 PMC11043046

[phy271080-bib-0009] Forhead, A. J. , & Fowden, A. L. (2014). Thyroid hormones in fetal growth and prepartum maturation. The Journal of Endocrinology, 221, R87–R103. 10.1530/JOE-14-0025 24648121

[phy271080-bib-0010] Gecgelen Cesur, M. , Cesur, G. , Ogrenim, M. , & Alkan, A. (2016). Do prenatal and postnatal hypothyroidism affect the craniofacial structure?: An experimental study. Angle Orthodontist, 86, 854–861. 10.2319/080315-521.1 26756376 PMC8597346

[phy271080-bib-0011] Ghanbari, M. , & Ghasemi, A. (2017). Maternal hypothyroidism: An overview of current experimental models. Life Sciences, 187, 1–8. 10.1016/j.lfs.2017.08.012 28807719

[phy271080-bib-0012] Hart, E. B. , & Steenbock, H. (1918). Thyroid hyperplasia and the relation of iodine to the hairless pig malady. I. Journal of Biological Chemistry, 33, 313–323. 10.1016/S0021-9258(18)86585-1

[phy271080-bib-0013] Ingram, D. L. , & Dauncey, M. J. (1986). Role of thyroid hormones in the modification of diet‐induced thermogenesis by propranolol. Hormone and Metabolic Research, 18, 677–679. 10.1055/s-2007-1012405 3781473

[phy271080-bib-0014] Innis, S. M. , Pasternak, J. A. , & Cabot, R. (2026). Fetal hypothyroidism induces sex‐specific epigenetic and transcriptomic alterations in the porcine placenta. Biology of Reproduction, 115, 233–249. 41795955 10.1093/biolre/ioag050

[phy271080-bib-0015] Ison, E. K. , Hopf‐Jannasch, A. S. , Harding, J. C. S. , & Alex Pasternak, J. (2022). Effects of porcine reproductive and respiratory syndrome virus (PRRSV) on thyroid hormone metabolism in the late gestation fetus. Veterinary Research, 53, 74. 10.1186/s13567-022-01092-3 36175938 PMC9524047

[phy271080-bib-0016] Ison, E. K. , Kent‐Dennis, C. E. , Fazioli, J. , Mulligan, M. K. , Pham, A. , & Pasternak, J. A. (2023). Compensatory mechanisms in response to induced hypothyroidism in the late gestation pig fetus†. Biology of Reproduction, 108, 731–743. 10.1093/biolre/ioad024 36811850 PMC10183360

[phy271080-bib-0017] Jeon, D. , Smith, A. , Cabot, R. , & Pasternak, J. A. (2026). Vertical transmission of PRRSV2 strain NVSL 97‐7895 occurs during mid‐gestation. Veterinary Microbiology, 318, 111043. 10.1016/j.vetmic.2026.111043 42035531

[phy271080-bib-0018] Jeon, D. , Smith, A. A. , Innis, S. , Cabot, B. , Cabot, R. , & Pasternak, J. A. (2025). Surgical alteration of uterine space influences embryonic loss and fetal growth in the contemporary pig. BMC Veterinary Research, 21, 360. 10.1186/s12917-025-04820-x 40390048 PMC12090578

[phy271080-bib-0019] Klein, R. Z. , Haddow, J. E. , Faix, J. D. , Brown, R. S. , Hermos, R. J. , Pulkkinen, A. , & Mitchell, M. L. (1991). Prevalence of thyroid deficiency in pregnant women. Clinical Endocrinology, 35, 41–46. 10.1111/j.1365-2265.1991.tb03494.x 1889138

[phy271080-bib-0020] Ko, H. , Sammons, J. , Pasternak, J. A. , Hamonic, G. , Starrak, G. , MacPhee, D. J. , Detmer, S. E. , Plastow, G. S. , & Harding, J. C. S. (2022). Phenotypic effect of a single nucleotide polymorphism on SSC7 on fetal outcomes in PRRSV‐2 infected gilts. Livestock Science, 255, 104800. 10.1016/j.livsci.2021.104800

[phy271080-bib-0021] Krysin, E. , Brzezińska‐Slebodzińska, E. , & Slebodziński, A. B. (1997). Divergent deiodination of thyroid hormones in the separated parts of the fetal and maternal placenta in pigs. The Journal of Endocrinology, 155, 295–303. 10.1677/joe.0.1550295 9415064

[phy271080-bib-0022] Lanham, S. A. , Fowden, A. L. , Roberts, C. , Cooper, C. , Oreffo, R. O. C. , & Forhead, A. J. (2011). Effects of hypothyroidism on the structure and mechanical properties of bone in the ovine fetus. The Journal of Endocrinology, 210, 189–198. 10.1530/JOE-11-0138 21642376

[phy271080-bib-0023] Lauffer, P. , Zwaveling‐Soonawala, N. , Naafs, J. C. , Boelen, A. , & van Trotsenburg, A. S. P. (2021). Diagnosis and Management of Central Congenital Hypothyroidism. Frontiers in Endocrinology (Lausanne), 12, 686317. 10.3389/fendo.2021.686317 PMC8458656 34566885

[phy271080-bib-0024] Leary, S. L. (2020). AVMA Guidelines for the Euthanasia of Animals: 2020 Edition (2020th ed.). American Veterinary Medical Association.

[phy271080-bib-0025] Lenth, R. V. , & Piaskowski, J. (2017). emmeans: Estimated Marginal Means, aka Least‐Squares Means. 2.0.3.

[phy271080-bib-0026] Lunney, J. K. , Van Goor, A. , Walker, K. E. , Hailstock, T. , Franklin, J. , & Dai, C. (2021). Importance of the pig as a human biomedical model. Science Translational Medicine, 13, eabd5758. 10.1126/scitranslmed.abd5758 34818055

[phy271080-bib-0027] Malgarin, C. M. , Moser, F. , Pasternak, J. A. , Hamonic, G. , Detmer, S. E. , MacPhee, D. J. , & Harding, J. C. S. (2021). Fetal hypoxia and apoptosis following maternal porcine reproductive and respiratory syndrome virus (PRRSV) infection. BMC Veterinary Research, 17, 182. 10.1186/s12917-021-02883-0 33933084 PMC8088663

[phy271080-bib-0028] Mallela, M. K. , Strobl, M. , Poulsen, R. R. , Wendler, C. C. , Booth, C. J. , & Rivkees, S. A. (2014). Evaluation of developmental toxicity of propylthiouracil and methimazole. Birth Defects Research. Part B, Developmental and Reproductive Toxicology, 101, 300–307. 10.1002/bdrb.21113 24980470 PMC4295064

[phy271080-bib-0029] McPherson, R. L. , Ji, F. , Wu, G. , Blanton, J. R. , & Kim, S. W. (2004). Growth and compositional changes of fetal tissues in pigs. Journal of Animal Science, 82, 2534–2540. 10.2527/2004.8292534x 15446468

[phy271080-bib-0030] Mulligan, M. K. , Kleiman, J. E. , Caldemeyer, A. C. , Harding, J. C. S. , & Pasternak, J. A. (2022). Porcine reproductive and respiratory virus 2 infection of the fetus results in multi‐organ cell cycle suppression. Veterinary Research, 53, 13. 10.1186/s13567-022-01030-3 35189966 PMC8860275

[phy271080-bib-0031] Nakamura, R. , Teshima, R. , Hachisuka, A. , Sato, Y. , Takagi, K. , Nakamura, R. , Woo, G.‐H. , Shibutani, M. , & Sawada, J. (2007). Effects of developmental hypothyroidism induced by maternal administration of methimazole or propylthiouracil on the immune system of rats. International Immunopharmacology, 7, 1630–1638. 10.1016/j.intimp.2007.08.012 17996672

[phy271080-bib-0032] National Research Council . (2012). Nutrient Requirements of Swine: Eleventh Revised Edition. National Academies Press.

[phy271080-bib-0033] Pasternak, J. A. , MacPhee, D. J. , & Harding, J. C. S. (2020). Maternal and fetal thyroid dysfunction following porcine reproductive and respiratory syndrome virus2 infection. Veterinary Research, 51, 47. 10.1186/s13567-020-00772-2 32228691 PMC7106657

[phy271080-bib-0034] Patriota, E. S. O. , Lima, I. C. C. , Nilson, E. A. F. , Franceschini, S. C. C. , Gonçalves, V. S. S. , & Pizato, N. (2022). Prevalence of insufficient iodine intake in pregnancy worldwide: A systematic review and meta‐analysis. European Journal of Clinical Nutrition, 76, 703–715. 10.1038/s41430-021-01006-0 34545212

[phy271080-bib-0035] Pinheiro, J. , Bates, D. , & R Core Team . (1999). nlme: Linear and Nonlinear Mixed Effects Models. 3.1‐169.

[phy271080-bib-0036] Rudy, K. G. (2024). Ontological Changes in the Swine Fetus and Placenta from Mid‐ to Late‐Gestation. Purdue University Graduate School: 1979271 Bytes.

[phy271080-bib-0037] Salisbury, S. (2003). Cretinism: The past, present and future of diagnosis and cure. Paediatrics & Child Health, 8, 105–106. 10.1093/pch/8.2.105 20019927 PMC2791432

[phy271080-bib-0038] Schneider, C. A. , Rasband, W. S. , & Eliceiri, K. W. (2012). NIH image to ImageJ: 25 years of image analysis. Nature Methods, 9, 671–675. 10.1038/nmeth.2089 22930834 PMC5554542

[phy271080-bib-0039] Smith, A. A. , Hamonic, G. , Plastow, G. S. , Harding, J. C. S. , & Pasternak, J. A. (2026). Hypothalamic‐pituitary‐thyroid and adrenal Axis modulation in response to fetal porcine reproductive and respiratory virus infection. Comprehensive Physiology, 16, e70112. 10.1002/cph4.70112 41663338 PMC12886161

[phy271080-bib-0040] Smith, A. A. , & Pasternak, J. A. (2025a). Porcine fetal hypothyroidism induces temporal and tissue‐specific alterations in the insulin‐like growth factor system. Comprehensive Physiology, 15, e70028. 10.1002/cph4.70028 40693299 PMC12281263

[phy271080-bib-0041] Smith, A. A. , & Pasternak, J. A. (2025b). Gestational age and fetal hypothyroidism Alter the porcine thyroid and local hepatic and renal renin‐angiotensin systems. Physiological Reports, 13, e70565. 10.14814/phy2.70565 40939099 PMC12431595

[phy271080-bib-0042] Smith, A. A. , Vesey, A. , Helfrich, C. , & Pasternak, J. A. (2024). Late gestation fetal hypothyroidism alters cell cycle regulation across multiple organ systems. BMC Veterinary Research, 20, 268. 10.1186/s12917-024-04102-y 38902754 PMC11188211

[phy271080-bib-0043] Wickham, H. (2016). ggplot2: Elegant Graphics for Data Analysis (2nd ed.). Springer International Publishing: Imprint: Springer.

